# Automating hybrid collective intelligence in open-ended medical diagnostics

**DOI:** 10.1073/pnas.2221473120

**Published:** 2023-08-14

**Authors:** Ralf H. J. M. Kurvers, Andrea Giovanni Nuzzolese, Alessandro Russo, Gioele Barabucci, Stefan M. Herzog, Vito Trianni

**Affiliations:** ^a^Center for Adaptive Rationality, Max Planck Institute for Human Development, Berlin 14191, Germany; ^b^Science of Intelligence, Research Cluster of Excellence, Berlin 10587, Germany; ^c^Semantic Technology Laboratory & Collective Intelligence in Natural and Artificial Systems Laboratory, Institute of Cognitive Sciences and Technologies, National Research Council, Rome 00185, Italy; ^d^Norwegian University of Science and Technology, Trondheim 7034, Norway

**Keywords:** collective intelligence, general medical diagnostics, ontology, natural language processing

## Abstract

Collective intelligence has emerged as a powerful mechanism to boost decision accuracy across many domains, such as geopolitical forecasting, investment, and medical diagnostics. However, collective intelligence has been mostly applied to relatively simple decision tasks (e.g., binary classifications). Applications in more open-ended tasks with a much larger problem space, such as emergency management or general medical diagnostics, are largely lacking, due to the challenge of integrating unstandardized inputs from different crowd members. Here, we present a fully automated approach for harnessing collective intelligence in the domain of general medical diagnostics. Our approach leverages semantic knowledge graphs, natural language processing, and the SNOMED CT medical ontology to overcome a major hurdle to collective intelligence in open-ended medical diagnostics, namely to identify the intended diagnosis from unstructured text. We tested our method on 1,333 medical cases diagnosed on a medical crowdsourcing platform: The Human Diagnosis Project. Each case was independently rated by ten diagnosticians. Comparing the diagnostic accuracy of single diagnosticians with the collective diagnosis of differently sized groups, we find that our method substantially increases diagnostic accuracy: While single diagnosticians achieved 46% accuracy, pooling the decisions of ten diagnosticians increased this to 76%. Improvements occurred across medical specialties, chief complaints, and diagnosticians’ tenure levels. Our results show the life-saving potential of tapping into the collective intelligence of the global medical community to reduce diagnostic errors and increase patient safety.

## Significance

In the United States, an estimated 250,000 people die annually from preventable medical errors, many of which originate during the diagnostic process. A powerful approach to increase diagnostic accuracy is to combine the diagnoses of multiple diagnosticians. However, we lack methods to aggregate independent diagnoses in general medical diagnostics. Using knowledge engineering methods, we introduce a fully automated solution to this problem. We tested our solution on 1,333 medical cases, each of which was independently diagnosed by ten diagnosticians. Our solution substantially increases diagnostic accuracy: Single diagnosticians achieved 46% accuracy, pooling the decisions of ten diagnosticians increased this to 76%. These results demonstrate that collective intelligence can reduce diagnostic errors, promoting health services and trust in the global medical community.

Collective intelligence (CI) has been shown to boost the accuracy of decisions across a wide range of domains, from geopolitical forecasting, to investment decisions and medical diagnostics (1–8). However, CI has been mostly applied to relatively simple decision-making tasks, with well-defined answer sets, such as binary or multiclass classification or continuous estimation tasks (9–12). Unlocking the potential of crowds for more complex tasks with a much larger answer set, such as emergency management or general medical diagnostics, has been much harder. The open-ended nature of question and answer formats presents a hard problem, as it is difficult to identify, label, and aggregate the incommensurable judgments from different experts (13).

Relying solely on algorithmic processes to solve such complex decision-making tasks is also challenging for at least two reasons. First, human decision-makers may be especially reluctant to trust purely algorithmic solutions in complex, open-ended decision tasks (14). Second, the computational complexity and the scale of the problem space may be too vast to be exhaustively explored by domain-agnostic algorithms—thus the need to incorporate human domain knowledge (15). In such high-dimensional problem spaces, human experts are often needed to guide the search process and to narrow down the set of possible solutions. To aid humans—and AI alike—in navigating the problem space, knowledge engineering approaches provide models to structure the various solutions (e.g., medical diagnoses) in a hierarchical manner, e.g., using ontologies for exploiting interrelationships between relevant concepts; (16, 17). Here, we show how one can leverage such knowledge representation models to harness CI in a complex decision-making task, overcoming some of the key challenges hampering CI in open-ended tasks. We illustrate this general approach in the domain of general medical diagnostics, that is, the problem of identifying the correct diagnosis for a patient out of a very large set of potential diagnoses.

Diagnostic errors are a leading cause of death in the United States (18–22). Apart from loss of life, diagnostic errors contribute to incorrect treatments, patient morbidity, opportunity costs in the efficient use of scarce resources, and erosion of trust in the healthcare system. CI is currently actively explored as a way to reduce diagnostic errors, by relying on the intelligence of multiple diagnosticians, rather than single diagnosticians—as is often medical practice. CI can arise via different mechanisms, such as aggregating the independent decisions of decision-makers, a.k.a., wisdom of crowd approaches (11, 23–25), group discussions (4, 26), or market mechanisms (1). Here, we focus on the wisdom of the crowd approach, which is a promising approach in medical diagnostics as it allows to gather judgments from diagnosticians worldwide without the need to coordinate efforts in time or space.

Previous research on CI in medical diagnostics has shown that pooling independent decisions of diagnosticians can substantially boost diagnostic accuracy. This has, however, predominantly been shown in well-defined, binary or multiclass classification tasks, such as mammography (27, 28), dermatology (29), low back pain diagnostics (30), and emergency medicine (31). There is little research on how to aggregate diagnoses in general medical diagnostics, where the diagnoses need to be selected from a very large number of possible diagnoses. In one notable exception, Barnett et al. (32) used data from a large medical crowdsourcing platform, the Human Diagnosis Project [Human Dx, https://www.humandx.org/, (33)] to study the aggregation of independent decisions in general medical diagnostics. Their results suggest that pooling independent diagnoses from multiple medical experts is a powerful mechanism to boost diagnostic accuracy in general medical diagnostics. Their approach has, however, four drawbacks which restricts its validity and usefulness. First, human experts were used to evaluate the accuracy of the provided diagnoses. These experts determined whether a diagnosis provided by a diagnostician matched the correct diagnosis of the medical case in question. This is a time-consuming procedure as each medical case requires several manual comparisons among the provided diagnoses. Moreover, this human intervention step introduces both the potential of disagreement among experts on how to best standardize terms and a range of unwanted coding biases because of the nonblinded coding (34). Second, this matching step was only done with respect to the correct diagnosis of a medical case. That is, synonyms of the correct diagnosis of a case were aggregated, whereas synonyms of reported diagnoses which were incorrect were not aggregated, providing an unfair aggregation advantage to the correct diagnosis. Third, this approach cannot harvest the vast domain knowledge that has been amassed in medical science, in particular, the interrelationships between different diagnoses as encoded in medical ontologies. Finally, this approach may be practical for training cases, where the correct diagnosis is known ahead of time, but not for actual clinical practice, where the correct diagnosis is not yet known.

Here, we develop and test a fully automated (i.e., not requiring human intervention), scalable procedure for employing CI in open-ended general medical diagnostics that exploits knowledge engineering techniques to take advantage of the structured domain knowledge available in medicine and healthcare. Addressing the above-mentioned drawbacks, we will show that our automated approach is able to harness CI across a range of group sizes, medical domains, and levels of expertise. Next, we will show how exploiting interrelationships among medical concepts unlocks a suite of possibilities for harnessing CI.

## Experimental Setup and Methods

### Background and Source Dataset.

**Fig. 1.**
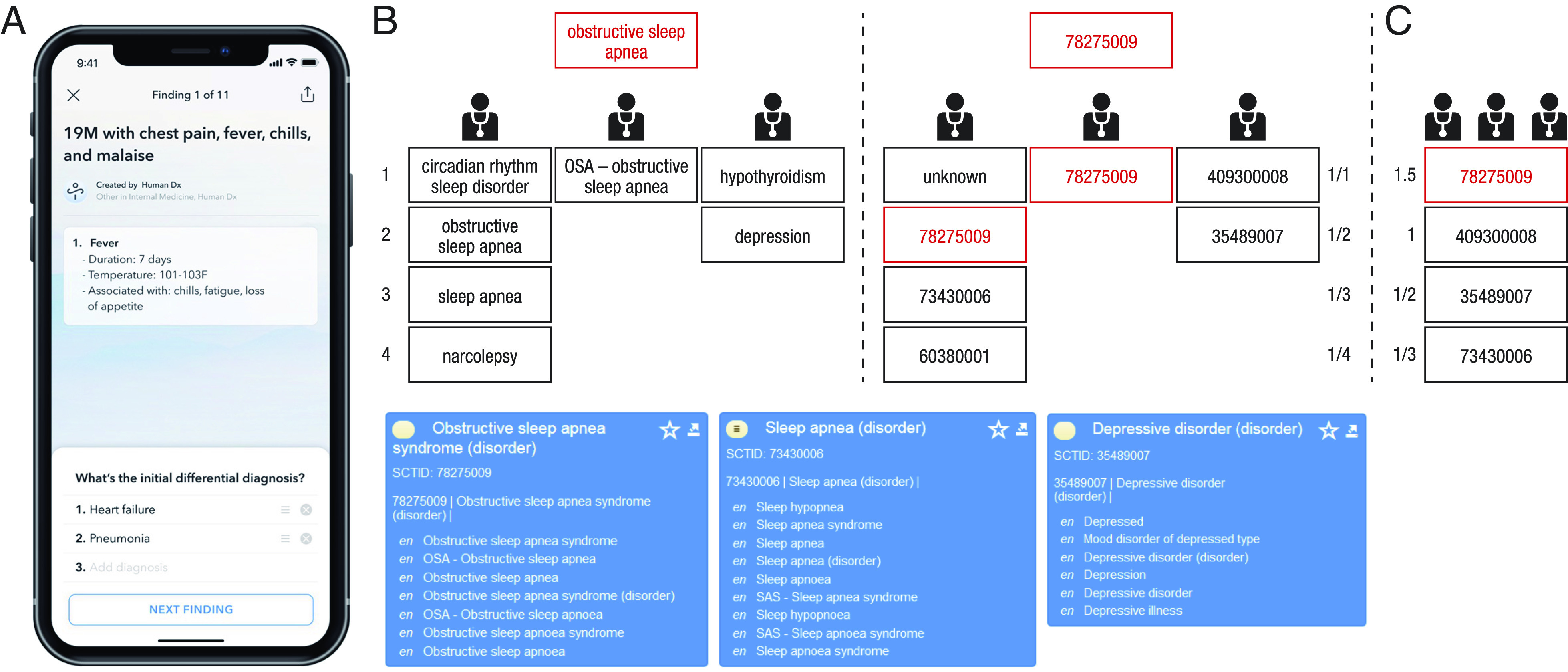
Illustration of the automated pipeline harnessing collective intelligence in open-ended medical diagnostics with the help of the medical ontology SNOMED Clinical Terms (*A*) Example of the first page of a medical case as shown to users accessing the Human Dx platform via a mobile device. The page contains general patient information, clinical findings, and the possibility to enter an initial differential diagnosis; for our analysis, we did not consider any of the initial differential diagnoses, but only the final diagnosis given by a user. Users can move to the next screen by clicking the “next finding” button. (*B*) Illustration of the mapping of different diagnoses to SNOMED CT identifiers (SCTIDs). The *Left* part shows the normalized diagnoses (i.e., the text strings after NLP normalization; see main text for details) of three users. Users 1, 2, and 3 provided four, one, and two diagnoses, respectively. The correct diagnosis of the case is shown in red for illustration, but is not used—or needed—for the aggregation process. All users’ diagnoses—and the correct diagnosis—were assigned to a SCTID using only exact word matches (i.e., a Jaccard similarity of 1 after NLP normalization; see main text for details). In this case, one diagnosis (“circadian rhythm sleep disorder”) could not be matched. The blue text boxes show three SCTIDs present in this example. The first box shows the correct SCTID 78275009 with its “Fully Specified Name” (FSN) “Obstructive sleep apnea syndrome.” Crucially, each SCTID contains a list of synonyms which all refer to the same SCTID (*SI Appendix*, Fig. S1), which includes the terms “obstructive sleep apnea” (used by user 1) and “OSA—obstructive sleep apnea” (used by user 2). Both terms are thus assigned to the same SCTID 78275009. The second and third box contain two incorrect SCTIDs, covering other diagnoses provided by users. (*C*) The collective ranking after aggregation. The collective support for each unique SCTID was determined using a 1/r scoring rule, where r is the rank of a diagnosis given by a user. The first diagnosis of a user received a score of 1/1, the second diagnosis 1/2, etc. (panel *B*). The scores for each unique SCTID were then summed, and the SCTIDs were sorted from the highest to lowest score. In this case, the SCTID 78275009 received a score of 1/2+1/1=1.5, which was the highest score; hence, it appeared at the top of the collective ranking.

Our approach uses a large dataset on general medical diagnostics collected by the Human Diagnosis Project (Human Dx). Human Dx is an online collaborative effort created to provide a global teaching environment for clinicians and to tap into the wisdom of the global medical community. It comprises an online platform to which medical experts can submit and solve patient cases. Patient cases consist of general patient information (e.g., age, gender, general symptoms) and a series of clinical findings, such as the outcomes of physical and diagnostic tests (e.g., laboratory and imaging studies; Fig. 1*A*). The medical experts creating the case know the correct diagnosis from further follow-up research. An expert panel reviews each case and decides whether the case is of sufficient quality and representative of the given domain. If so, the case is published and becomes accessible to the users of the platform. Cases may be removed from the platform if many users indicate that a case is problematic in terms of clarity or quality. Human Dx tags cases with a label indicating the prime specialty of a case, with cases stemming from a wide range of medical specialties (e.g., cardiology, dermatology, endocrinology, neurology, etc.).

Users from all over the world are invited to register at the platform and diagnose the uploaded medical cases. A case starts with showing general patient information and the first clinical findings (Fig. 1*A*). A user can proceed to the next finding of a case by button click. After observing all findings, they are asked to submit their diagnosis. The user can provide a single diagnosis or a ranked list of multiple diagnoses. Moreover, they can enter each diagnosis as free text or select an option from a catalog of medical terms (suggested as they type). After submitting their diagnosis, the user receives the correct solution of the case.

We analyzed all cases created between May 7, 2014, through October 5, 2016, for which at least 10 users provided a diagnosis (1,572 cases). For each case, 10 diagnosticians were randomly sampled from all diagnosticians who completed at least one other case before. In this way, we select only diagnosticians with some experience in using the platform. The sample included 2,069 unique users from 47 different countries, though predominantly from the United States (91%). We used self-reported tenure to determine the seniority of users (medical student, intern, resident, fellow, attending physician).

### An Automatic, Reproducible, and Scalable Method to Identify Exact Medical Concepts from Free-Text Diagnoses.

Arguably, the biggest challenge for the aggregation of independent diagnoses in open-ended medical diagnostics is to identify which diagnoses point to the same medical concept. This includes mundane challenges (e.g., British versus American spelling, capitalized letters or not, punctuation, typos, etc.) but also the thorny problem of determining whether or not two reported diagnoses are equivalent. To address these issues, we developed an automatic, reproducible, and scalable method to identify exact medical concepts from free-text diagnoses, which relies on a combination of semantic knowledge graphs and natural language processing (NLP) and integrates a publicly available medical ontology, i.e., the SNOMED Clinical Terms ontology (SNOMED CT). SNOMED CT is a systematically organized computer-processable collection of medical terms and considered the most comprehensive, multilingual clinical healthcare terminology in the world (35, 36) containing over 78,000 unique diseases (37). Specifically, our method leverages a semantic knowledge graph that we constructed by applying knowledge engineering techniques in order to reuse design best practices, e.g., ontology design patterns; (38) and linking information about medical cases and users’ diagnoses to SNOMED CT, which we exploit by gathering definitions for and taxonomic relations among clinical terms (*Material and Methods* and *SI Appendix*).

Fig. 1*B* illustrates our knowledge engineering approach. The knowledge graph is constructed with data from the Human Dx dataset, using knowledge-engineering techniques to extract the knowledge available from the dataset in terms of relations among concepts and subject–predicate–object triples (*SI Appendix*), matching the concepts related to clinical terms against the SNOMED CT ontology, which is therefore aligned within our knowledge graph. This is a two-step process. The first step is string normalization, whereby we use routine NLP tools to standardize all diagnoses (both the ones given by the users and the correct ones provided by a case’s author). This step consists of a series of text normalization procedures, including removing stop words, converting British English to US English, converting plural to singular, and identification of acronyms (see *Material and Methods* and *SI Appendix* for a complete description of the procedure). In the second step (concept mapping), we mapped each of these normalized diagnoses to an existing medical concept within SNOMED CT (July 2020 International Edition Release). For each diagnosis, we identified which SNOMED CT identifier(s) (SCTID) exactly matched a normalized diagnosis. To illustrate, the character strings “obstructive sleep apnea,” “osa,” “OSA,” and “OSA—obstructive sleep apnea” are all considered synonyms pointing to the same SCTID 78275009 (*SI Appendix*, Fig. S1). The character string “sleep apnea,” however, points to a different SCTID (73430006; see also Fig. 1*B*). We considered only exact word matches (i.e., a Jaccard similarity of 1, see *Materials and Methods*; in *SI Appendix*, we show additional analyses relaxing this matching criterion). Occasionally, a character string showed an exact match with more than one SCTID (4.4% for correct diagnoses and 5.0% for users’ diagnoses). For example, the character string “Kaposi’s sarcoma” returned two SCTIDs named “Kaposi’s sarcoma (disorder)” and “Kaposi’s sarcoma, morphology (morphologic abnormality),” respectively. In such cases, we relied on the semantic tags of the SCTID to identify the most likely correct match. Semantic tags indicate where a concept fits into the medical hierarchy (i.e., disorder, finding, morphological abnormality, body structure, person, organism, or specimen). Since our primary goal is to identify diagnoses, we selected the SCTID in the following order: disorder, finding, morphological abnormality, and organism. This approach is corroborated by the observation that for all situations in which the matching of a case’s correct diagnosis returned only one SCTID (which happened in 95.6% of cases), these SCTIDs where overwhelmingly disorders (96.8%), followed by findings (2.0%), morphological abnormality (1.0%), and organism (0.2%).

We first applied this pipeline to the correct diagnoses of all the 1,572 cases. After normalizing the correct diagnoses, our approach could exactly match 1,333 (84.8%) correct diagnoses to an SCTID (*SI Appendix*, Fig. S2*A*). For these cases, we thus can be certain that we have identified the correct solution according to SNOMED CT. In the remainder, we focus on this set of 1,333 cases. The conservative approach of using perfect matching assures that we do not introduce errors when assigning a (correct) diagnosis to a SCTID. Next, we applied our approach to the diagnoses provided by the users who solved these 1,333 cases: 41,242 (out of 47,772; 86,9%; *SI Appendix*, Fig. S2*B*) could be exactly matched to a SCTID. The remaining 13.1% remained unidentified and were discarded from the analyses.

## Results

### Aggregating Independent Diagnoses in General Medical Diagnostics.

Having mapped the correct diagnoses and the users’ diagnoses into an integrated knowledge graph allows us to automatically aggregate users’ diagnoses and test how collective diagnoses compare to individual ones in terms of diagnostic accuracy. For each of the 1,333 cases, we implemented the following procedure. We considered groups of varying sizes (1 to 10). For each group size, we created all possible unique groups. For each of these groups, we determined the collective support for each of the unique SCTIDs provided by the group members using three aggregation rules (where r is the rank of a diagnosis): 1/r, $1/r^{2}$, and equal-weighting rule. The 1/r rule (Fig. 1*C*) weighs a diagnosis by the inverse of r: The first diagnosis provided by a user (r=1) receives a score of 1, the second one (r=2) a score of 1/2, etc. The $1/r^{2}$ rule down-weighs diagnoses lower in the rank order more heavily (e.g., second-ranked diagnosis receives a score of 1/4). The equal-weighting rule weighs all diagnoses equally (i.e., independent of order).

Within each group–and for each scoring rule—we summed the score for each of the unique SCTIDs provided by the group members and ranked the SCTIDs from the highest to lowest score. In case of tied scores, we ordered SCTIDs according to their semantic tags (in the same order as used in the concept mapping step, i.e., disorder, finding, morphological abnormality, and organism). If SCTIDs were still tied, we randomized the order within the respective tied SCTIDs. Finally, we determined whether the correct SCTID was present in the top 1, 2, or 3 diagnoses in the collective ranking.

**Fig. 2.**
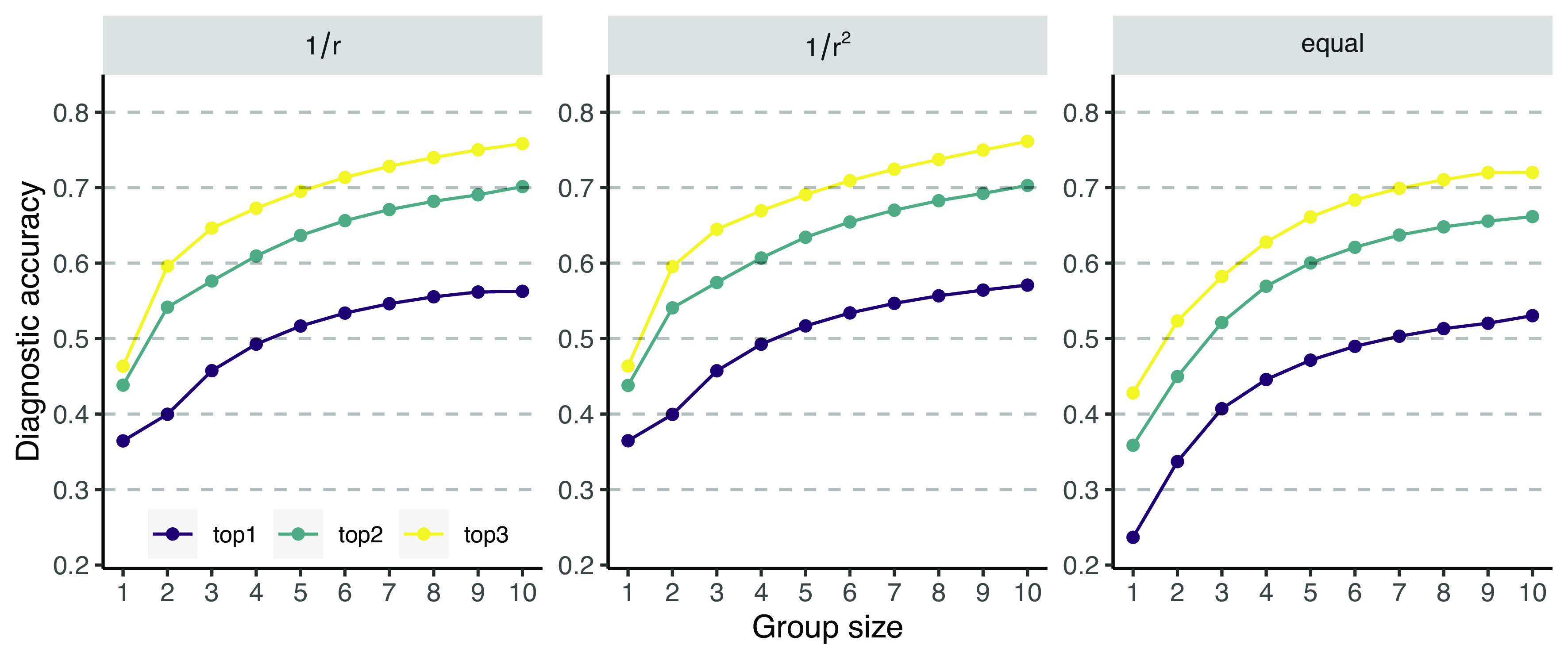
Diagnostic accuracy of the fully automated aggregation procedure for different group sizes for three different aggregation rules. Increasing the number of group members increases the likelihood that the correct diagnosis is present in the top 1, top 2, or top 3 of the collective diagnosis when using a 1/r, $1/r^{2}$, or equal-weighting rule, respectively.

Fig. 2 presents the results of this automated aggregation procedure, showing the average performance at each group size across all cases. Increasing the number of group members increases the diagnostic accuracy, that is, the likelihood that the correct diagnosis is present among the top 1, top 2, or top 3 of the collective ranking. For example, the likelihood that the correct diagnosis is present in the top 3 of the collective ranking increases from 46% for singletons to 76% for groups of 10 diagnosticians under the 1/r rule (Fig. 2, *Left*). The other two aggregation rules also lead to an increase in diagnostic accuracy with increasing group size (Fig. 2, *Center* and *Right*). There is, however, a difference in how much the diagnostic accuracy is increased. The equal-weighing rule (Fig. 2, *Right*) generally performed worse than both other rules, suggesting that the rank order of a diagnosis in a user’s diagnosis positively predicts diagnostic accuracy. *SI Appendix*, Fig. S3 shows that this indeed was the case: The diagnosis ranked first by a user was much more likely to be correct than lower-ranked diagnoses. These results indicate that it is important to give more weight to first-ranked diagnoses (as compared to equal weighing) but that the exact strength of this upweighing is less important. In the following, we will focus on the 1/r rule.

**Fig. 3.**
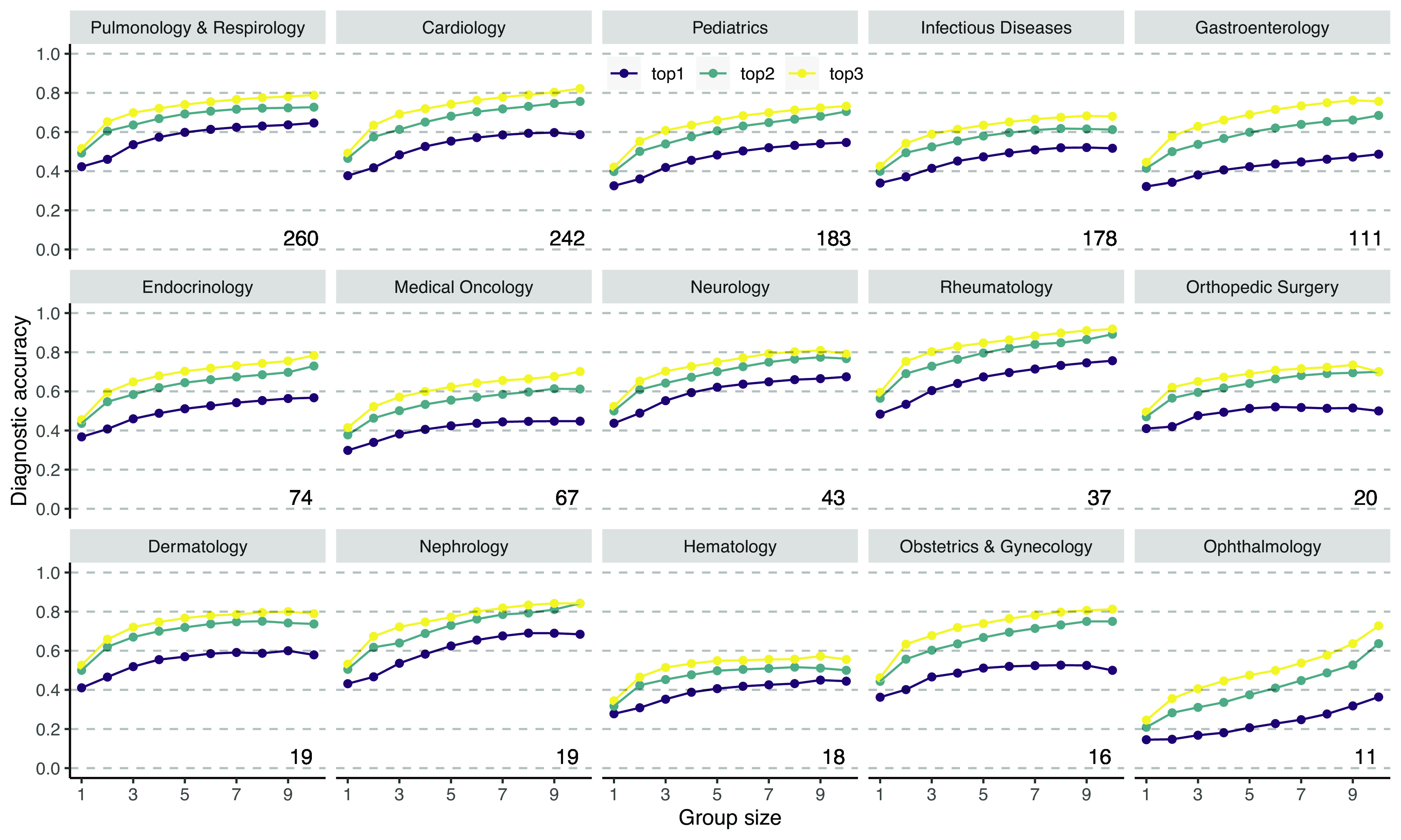
Diagnostic accuracy of the aggregation procedure for different group sizes for different medical specialties of the cases using the 1/r rule. Across medical specialties, increasing the number of group members increases the likelihood that the correct diagnosis is present in the top 1, top 2, or top 3 of the collective diagnosis. Numbers at the *Bottom Right* indicate the number of cases within that specialty. Medical specialties are ordered from the highest to lowest number of cases.

**Fig. 4.**
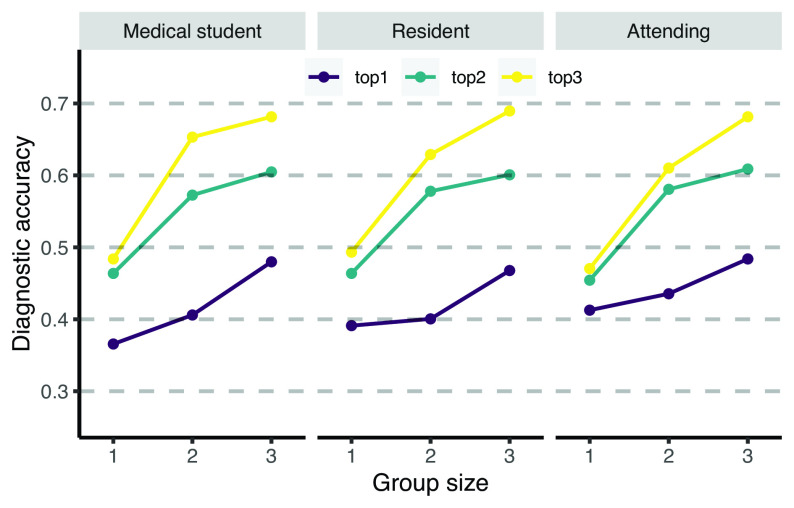
Diagnostic accuracy of the aggregation procedure for different group sizes for different tenure levels of users using the 1/r rule. Across different tenure levels, increasing the number of group members increases the likelihood that the correct diagnosis is present in the top 1, top 2, or top 3 of the collective diagnosis.

Next, we investigated whether improvements in diagnostic accuracy are robustly present across different medical specialties of cases (only considering medical specialties with more than 10 cases). Fig. 3 shows the diagnostic accuracy for different group sizes per medical specialty, showing that the improvement in diagnostic accuracy with group size is robustly found across medical specialties. *SI Appendix*, Fig. S4 shows that the same holds across different chief complaints of cases. Next, we compared different tenure levels. Of the 13,330 unique diagnoses, 3,054 were given by medical students (23%), 1,352 by interns (10%), 5,340 by residents (40%), 179 by fellows (1%), and 3,405 by attending physicians (26%). We compared the performance of small groups across the three most prevalent tenure levels. We considered only cases which were completed by at least three medical students, three residents, and three attending physicians (n=62 cases). Note that such a strict within-cases comparison is required as a between-cases comparison may be confounded with self-selection of users into cases. We used the same simulation procedure as described earlier. Fig. 4 shows that small groups outperformed single diagnosticians across all tenure levels. Attending physicians performed slightly better than medical students and residents when considering whether the correct diagnosis was ranked first, but not when considering the top 2 or top 3 diagnoses. *SI Appendix*, Fig. S5 shows the results when including all cases which were completed by at least two medical students, two residents, and two attending physicians (*n* = 450 cases) showing largely similar results. To summarize, combining the independent diagnoses of multiple diagnosticians robustly increases diagnostic accuracy across medical case specialties, cases’ chief complaints, and diagnosticians’ tenure level.

### Exploiting the Interrelations of Concepts at the Collective Level.

**Fig. 5.**
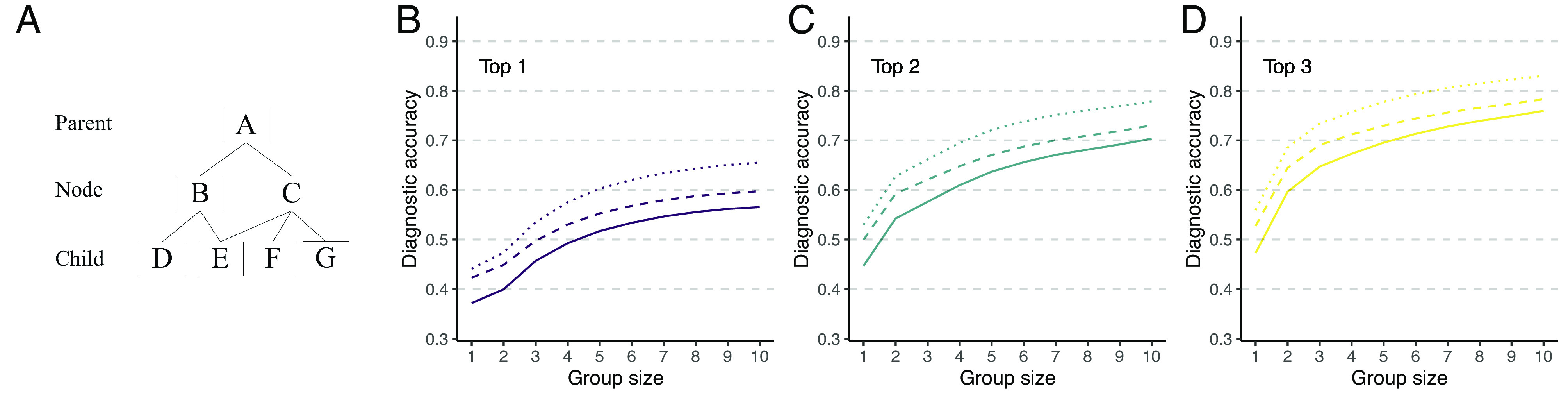
Diagnostic accuracy considering parent and child nodes using the 1/*r* rule. (*A*) SNOMED CT is organized as a polyhierarchy in which child nodes may have more than one parent nodes. (*B*–*D*) Diagnostic accuracy when only considering the correct node as correct solution (solid line), when also considering the parent node(s) of the correct note as correct solution (dotted line), or when also considering the child node(s) of the correct node as correct solution (dashed lines). Panels *B*–*D* show the results for considering whether the correct or connected diagnoses are in the top 1, top 2, or top 3 of the collective diagnosis, respectively (see inline titles).

Besides linking the correct diagnoses and the users’ diagnoses to existing concepts in SNOMED CT for the automatic aggregation of identical concepts, our knowledge engineering approach also allows extracting and capitalizing on the interrelationships between concepts in the knowledge graph. SNOMED CT concepts are organized in a polyhierarchy, a graph structure whereby concepts (a.k.a., nodes) are connected to (one or multiple) supertype “parent” and/or subtype “child” concepts (Fig. 5*A*). Our knowledge graph incorporates these semantic relations, so that a diagnosis without an identical match to the correct diagnosis can be associated with a parent or child concept of the correct diagnosis. Such a diagnosis is, typically, more relevant than a diagnosis that is neither a parent or child because the former is closer to the correct solution—in terms of network path distance—and has a higher likelihood of implying similar (or even identical) treatment recommendations (35). Therefore, we next explored diagnostic accuracy when exploiting these interrelationships. Fig. 5*B*–*D* shows how diagnostic accuracy scales with group size when we consider a diagnosis correct when it is either a i) direct match with the correct SCTID (as reported above), ii) child concept of the correct SCTID, or iii) parent concept of the correct SCTID. This approach substantially boosts diagnostic accuracy across all group sizes, as compared to only considering identical matches. This indicates that users’ frequently reported parent and/or child concepts of the correct diagnoses. *SI Appendix*, Fig. S6 shows how often users reported parent and child concepts of the correct diagnosis, showing that both parent and child concepts appeared regularly in single users, and increasingly so in higher-ranked positions, explaining the increased performance when considering these as correct responses.

## Discussion

This work presents a fully automated pipeline—spanning from the aggregation of diagnoses to the evaluation of the results obtained via CI—that can harness the power of independent medical experts in the medical domain at large. This thus vastly extends the application of CI in medical diagnostics beyond simple binary or multiclass classification or numeric estimation tasks. By integrating the correct diagnoses of cases and the users’ diagnoses into a semantic knowledge graph linked to a publicly available medical ontology (i.e., SNOMED CT), we were able to automatically aggregate the diagnoses of multiple users and compare how different group sizes and different aggregation rules fared against single users. Our results show that aggregation of independent responses from multiple users leads to substantial improvements in diagnostic accuracy across aggregation rules, medical specialties, chief complaints, and tenure levels of users.

A key contribution of our work is that our aggregation and evaluation procedures are fully automated, that is, do not require any manual, human intervention, [e.g., no need for manual mapping of free-text inputs to the correct solution by expert raters, as done in ref. 32] and can automatically identify synonyms unlike in ref. 39. This removes the human from the loop, avoiding the drawbacks and the possible biases of previous approaches, and allowing to scale up in a more time- and cost-efficient manner. Importantly, because the aggregation pipeline is fully automated and neither needs manual intervention or knowledge about the ground truth at the time of aggregation, it can operate in an actual, real-time clinical setting, where the ground truth is unknown at the time of judgments.

An important limitation of our study is the issue of representative design. Our results were obtained on a relatively large number of cases, but these cases were selected by an expert panel of Human Dx. Likewise, users can flag suspicious cases which may lead to their removal from the platform. As such, our results need to be understood within the current case selection procedure which may have, for example, selected against very difficult or rare cases. Future work should consider more ecologically valid ways of testing cases (40). Moreover, future work could study whether our method, next to arriving more often at the exact correct solution, also alters the likelihood of arriving at potentially beneficial (or harmful) diagnoses in terms of implied treatment. Finally, all our results are based on textual data in English. Possible next steps could be to generalize to other languages and even integrate diagnoses written in different languages, something that is made possible by the use of multilanguage ontologies such as SNOMED CT.

Future work—and (medical) crowdsourcing platforms—could explore the possibility of integrating other medical ontologies next to SNOMED CT (41). Combining different ontologies may further help in identifying the diagnoses provided by users and reduce the number of unidentified diagnoses. Integrating different ontologies may, however, be challenging, especially when they do not use a common terminology (42). For designing future medical crowdsourcing platforms (or other areas in which comprehensive ontologies have been developed), it may thus be advisable to rely on a single comprehensive ontology when eliciting users’ responses (e.g., when offering autocompletions). Starting from the onset with one comprehensive ontology and allowing users to only select from the realm of possibilities provided by an existing ontology will greatly simplify the subsequent collective aggregation of users’ responses. This, however, is only feasible when comprehensive ontologies exist, but given the key importance of ontologies in a diverse range of disciplines (16, 17), this design principle seems broadly applicable. However, the computational benefits of limiting users’ response options need to be traded off against a possible reduction in users’ engagement on a platform. To improve this trade-off, advanced methods of user interactions with complex ontologies could be implemented. When current ontologies in a domain are not fully developed, users could be allowed to add additional elements to existing ontologies, if they believe their idea is not captured by the extant knowledge structure. Other users could, in turn, be asked to verify these additions, and this would allow an iterative process between the platform and its users, and, in an ideal case, increase users’ motivation to contribute to the system, while simultaneously allowing the system to self-organize, adapt, and evolve (8, 43).

Future work could further explore the interrelations between concepts. More sophisticated approaches from network science could be employed to identify which diagnoses are closely related and capitalize on these insights e.g., bipartite graphs: ref. 44. As next steps, it could also be investigated whether collective performance can be further boosted by weighing users’ diagnoses according to their accuracy (45), expertise (46), similarity (47), or cognitive style (48). Furthermore, future work should incorporate insights and methods from information retrieval research and cognitive science on how to aggregate and evaluate lists of retrieval results (49–51).

Finally, other forms of collective intelligence which go beyond the wisdom of crowd approaches, such as consensus decision-making or combined decision-making (52, 53), could be investigated. Here, one could investigate leveraging individual heterogeneity and accuracy, how this interacts with case difficulty, and more broadly the process of social influence in open-ended domains.

## Supplementary Material

Appendix 01 (PDF)

## Data Availability

The code for running the aggregation simulations is uploaded on OSF: https://osf.io/h9qep/ (54). One Human Dx case is included to illustrate our approach. The full dataset with the collection of Human Dx cases we used in this experiment cannot be shared publicly because of privacy and data protection regulations but can be obtained by reaching out to Human Dx. The ontology (https://github.com/anuzzolese/crome/blob/main/crome-ontology.owl) (55), the RML mapping (https://github.com/anuzzolese/crome/blob/main/matching_map.ttl) (56), and the code we used for generating the knowledge graph for normalizing text (https://github.com/anuzzolese/crome/blob/main/convert.py) (57) are publicly available on GitHub.
