# Supplementary material for: Automating hybrid collective intelligence in open-ended medical diagnostics
Source: Proc Natl Acad Sci U S A. 2023 Aug 14;120(34):e2221473120. doi: 10.1073/pnas.2221473120 (PMC10450668; doi:10.1073/pnas.2221473120)
Supplement: Supplementary file 1 — Appendix 01 (PDF) [file pnas.2221473120.sapp.pdf]

Supplementary Information for

Automating Hybrid Collective Intelligence in Open-Ended Medical Diagnostics

Ralf H.J.M. Kurvers, Andrea Nuzzolese, Alessandro Russo, Gioele Barabucci, Stefan M. Herzog, Vito Trianni

Ralf H.J.M. Kurvers
E-mail: kurvers@mpib-berlin.mpg.de

This PDF file includes:

Supplementary text
Figs. S1 to S10
Table S1
SI References

Supporting Information Text

Dataset

Table S1 provides descriptive statistics for the input dataset from Human Dx. These data were used to construct a semantic knowledge graph (KG).

Table S1. Summary of input dataset.

Variable	*N*
Patient cases	1,572
Total users' diagnoses	47,472
Median users' diagnoses per case	30
Distinct correct diagnoses of cases (ground truth)	718
Unique users	2,069

Building a semantic knowledge graph

The semantic knowledge graph (KG) includes an OWL[*] ontology and an RDF[†] linked dataset. The OWL ontology represents the terminological component (T-box) and provides a formal schema for representing knowledge about the clinical cases, with their associated details, such as diagnoses, diseases, diagnostic procedures, patients' details, etc. The RDF linked dataset provides factual data (i.e., the A-box) by means of RDF triples modelled according to the schema formalised by the ontology. The KG is further enriched by providing formal linking to SNOMED Clinical Terms (SNOMED CT), a systematically-organized computer-processable collection of medical terms and considered the most comprehensive, multilingual clinical healthcare terminology in the world (1, 2).

The method for constructing the KG is reported in Figure S8, depicting a workflow using the UML notation. The methodology incorporates recent insights into best practices for KG construction (3, 4) and takes inspiration from previous projects including ArCo (5) and ScholarlyData (6). The methodology consists of the following three activities detailed below: ontology design, mapping-based KG population, and KG enrichment with SNOMED CT.

Ontology design. Figure S9 shows the ontology diagram. The diagram can be read from the concept `:Case`, which represents a medical case and is connected to a `:Patient`, a `:CaseCategory` (e.g., internal medicine, surgery, etc.), and a `:DiagnosisCollection`. A `:DiagnosisCollection` represents a ranked list of different diagnoses proposed by a user (i.e., a `:DiagnosingAgent`). Each `:Diagnosis` is grouped into `:DiagnosisCollection` using the class `:DiagnosisCollectionItem` as a container. We keep the notion of a diagnosis (i.e., a user identifies a disease as a possible solution for a medical case) separate from the disease itself. Hence, the object property `:ofDisease` is meant for associating a `:Diagnosis` with a `:Disease` in the KG. The concepts of the ontology are aligned with standard classes for enabling interoperability by means of a foundational ontology, i.e. DOLCE+DnS Ultralite[‡] (7).

Mapping-based knowledge graph population. The second activity (cf. Figure S8) populates the KG with facts (i.e., RDF triples) compliant with the ontology resulting from the previous activity. The KG population was performed by applying a mapping-based solution that relies on the RDF Mapping Language (RML) (8). RML is a language for expressing customized mappings from heterogeneous data structures and serialisations (e.g., CSV, TSV, XML and JSON) to RDF. RML is defined as a superset of the W3C-standardized mapping language R2RML[§] aimed at extending its applicability by adding support for data in other structured formats. A mapping describes how existing data can be converted to each component of an RDF triple, that is, subject–predicate–object, which is at the core of the RDF data model. We opted for the implementation of RML based on pyRML[¶], which is a lightweight RML engine written in Python.

Knowledge graph enrichment with SNOMED CT. The third activity enriches the KG with links to SNOMED CT. We constrained the linking to SNOMED CT to only the instances of the class `:Disease` in our KG. The goal is to identify possible matches and establish links between the diseases defined as diagnoses for the medical cases and the concepts defined in SNOMED CT. The linking with SNOMED CT was enabled by the LInk discovery framework for MEtric Spaces[‖] (LIMES) (9) tool. LIMES is a link-discovery framework for the Web of Data. It implements time-efficient approaches for large-scale link discovery based on the characteristics of different metric spaces.

[*] https://www.w3.org/TR/2012/REC-owl2-overview-20121211
[†] https://www.w3.org/TR/rdf-concepts/
[‡] http://www.ontologydesignpatterns.org/ont/dul/DUL.owl
[§] https://www.w3.org/TR/r2rml/
[¶] https://github.com/anuzzolese/pyrml
[‖] https://aksw.org/Projects/LIMES.html

Before linking the KG with SNOMED CT we first normalized the strings (representing the labels for diagnoses and SNOMED CT concepts) using standard Natural Language Processing (NLP) techniques. This was done to account for non-essential variations in the text inputs (uppercase vs. lowercase, British vs. US English etc.) and thus increase the likelihood that concepts are correctly matched. More specifically, we used the NORM[**] pipeline, which is part of LEXICAL TOOLS[††] and managed by the US National Library of Medicine.

> [NORM] creates an abstract representation of text strings allowing users to ignore alphabetic case, inflection, spelling variants, punctuation, genitive markers, stop words, diacritics, symbols, ligatures, and word order. The normalized string is a version of the original string in lower case, without punctuation, genitive markers, or stop words, diacritics, ligatures, with each word in its uninflected (citation) form, the words sorted in alphabetical order, and normalize non-ASCII Unicode characters to ASCII by mapping punctuation and symbols to ASCII, mapping Unicode to ASCII, stripping diacritics, splitting ligatures, and stripping non-ASCII Unicode characters.

More specifically, the following 11 normalization steps are carried out:

1. Q0: map Unicode symbols and punctuation to ASCII,
2. G: remove genitives,
3. RS: then remove parenthetic plural forms of (s), (es), (ies), (S), (ES), and (IES),
4. O: then replace punctuation with spaces,
5. T: then remove stop words,
6. L: then lowercase,
7. B: then uninflect each word,
8. CT: then get citation form for each base form,
9. Q7: then Unicode Core Norm
   - map Unicode symbols and punctuation to ASCII
   - map Unicode to ASCII
   - split ligatures
   - strip diacritics
10. Q8: then strip or map non-ASCII Unicode characters,
11. W: sort the words in alphabetic order.

Because diagnosticians commonly refer to clinical concepts using their acronyms, we extended the SNOMED CT terminology by deriving and adding acronyms for clinical concepts. This step aims at increasing the matching between elicited diagnoses and SNOMED CT concepts. To derive and add acronyms for SNOMED CT concepts, we exploited the naming conventions used for abbreviations and acronyms in the existing labels and synonyms[‡‡] to design regular expressions for extracting acronyms. For example, from the label "OSA - Obstructive sleep apnea" the acronym "OSA" is extracted and added to the corresponding concept. To avoid introducing ambiguities in the matching and linking process, an acronym was only added for a SNOMED CT concept if that acronym did not already exist for another concept.

We then used the LIMES framework to align diseases in our KG to the clinical terms in SNOMED CT by using the acronyms and normalized labels for computing the Jaccard similarity. For each disease associated with a diagnosis, we identified which SNOMED CT identifier(s) (SCTID) matched a normalized diagnosis with Jaccard similarity above a given threshold. Exact matches correspond to a Jaccard similarity of 1. The full KG is the output of this last matching step.

Figure S10 shows a sample of the final KG when applying this methodology. In this instantiation, the instance `:4233`, which is an instance of the class `:Case`, is linked to the instance `:4233_39`, which represents a collection of diagnosis solutions being an instance of the class `:DiagnosisCollection`. Such a collection contains ordered items among which the instance `:4233_39` is ranked 5th. The content of this item is the instance `:Differential_diagnosis_by_X_for_arteritis`, which is a `:Diagnosis` made by a certain user (i.e. `:agent_X`) for `:Arteritis`. The latter is an instance of the class `:Disease` and is linked to the clinical term with SCTID 520890018 which represents the "arteritis disorder" in SNOMED CT.

While the example in Fig. S10 shows an exact match between a disease in our KG and a SNOMED CT concept (expressed with a `owl:sameAs` relationship), we have also designed and instantiated (as part of the overall KG) a more general ontological model for representing matches between diseases and SNOMED CT concepts other than diseases (e.g., disorder, finding, morphological abnormality, body structure, person, organism, or specimen). In a nutshell, the general model allows representing a matching (as identified by the LIMES framework) in terms of:

[**] https://lhncbc.nlm.nih.gov/LSG/Projects/lvg/current/docs/userDoc/tools/norm.html
[††] https://lhncbc.nlm.nih.gov/LSG/Projects/lvg/current/web/index.html
[‡‡] https://confluence.ihtsdotools.org/display/DOCEG/General+Naming+Conventions

(i) the two entities involved in the matching, reporting for each of them details such as the normalized label resulting in a match and the property that originated the normalized string (e.g., `rdfs:label`, `skos:prefLabel` or `skos:altLabel` for a SNOMED CT concept);

(ii) the similarity metric used to evaluate the matching (in our setting the Jaccard similarity); and

(iii) the computed value for the similarity metric.

This general model allowed us to consider matches with a Jaccard similarity less than 1 and thus enabled additional analyses with a more lenient matching criterion (see the next section).

Results for relaxing exact matches

For the concept mapping in the main text, we only considered identical matches between users' normalized diagnoses and SNOMED CT Identifiers (SCTIDs). This was formalized as requiring a Jaccard similarity of 1 between users' diagnosis and SCTIDs. Indeed, a Jaccard similarity below 1 allows for the possibility of introducing errors when assigning a diagnosis to an SCTID and we did not want to create a situation in which the correct diagnosis was incorrectly identified. Lowering the threshold for the minimum Jaccard similarity value below 1 may have two effects with opposing expected consequences for diagnostic accuracy. On the one hand, a threshold below 1 may increase the frequency of users' diagnoses being assigned to an SCTID. Using a value of 1 led to a match in 86,9% of all users' diagnoses, meaning 13,1% of users' diagnoses were left unassigned. Increasing the amount of assigned users' diagnoses to an SCTID may increase individual and collective diagnostic accuracy because it can increase the number of valid judgments that effectively enter the aggregation (especially when small deviations, such as spelling errors or typos were preventing an exact match; that is, whenever they were not covered by our normalization pipeline). However, Jaccard similarity threshold values below 1 may also introduce more errors when linking a user's diagnosis to their intended SCTID concept thus reducing individual and collective diagnostic accuracy.

We performed additional analyses relaxing this strict threshold when matching users' diagnoses to a SCTID. For the correct diagnoses, we kept the strict threshold of 1. This was done because we wanted to compare the results to the same set of 1,333 cases (i.e., obtained when using a JI of 1). Otherwise, these simulations exactly followed the simulations reported in the main text, that is, we first ranked SCTIDs based on their collective support (using the three different scoring rules). In case of tied scores, we ordered SCTIDs according to their semantic tags (in the order: disorder, finding, morphological abnormality, organism), and when SCTIDs were still tied, we randomized the order within the respective tied SCTIDs. SI Appendix, Fig S7 shows how diagnostic accuracy scales with group size when including all terms with a Jaccard similarity value of 0.6 and higher. As can be seen, this substantially reduced diagnostic accuracy at all group sizes (though maintaining the positive effect of increasing group size). This is most likely the result of introducing increasingly more errors when assigning users' diagnoses to a SCTID. Lower values led to even worse performance. Also thresholds above 0.6 did not improve performance compared to a threshold of 1. Taken together, this suggests that a strict threshold of 1 is best suited to harness collective intelligence in open-ended medical diagnostics.

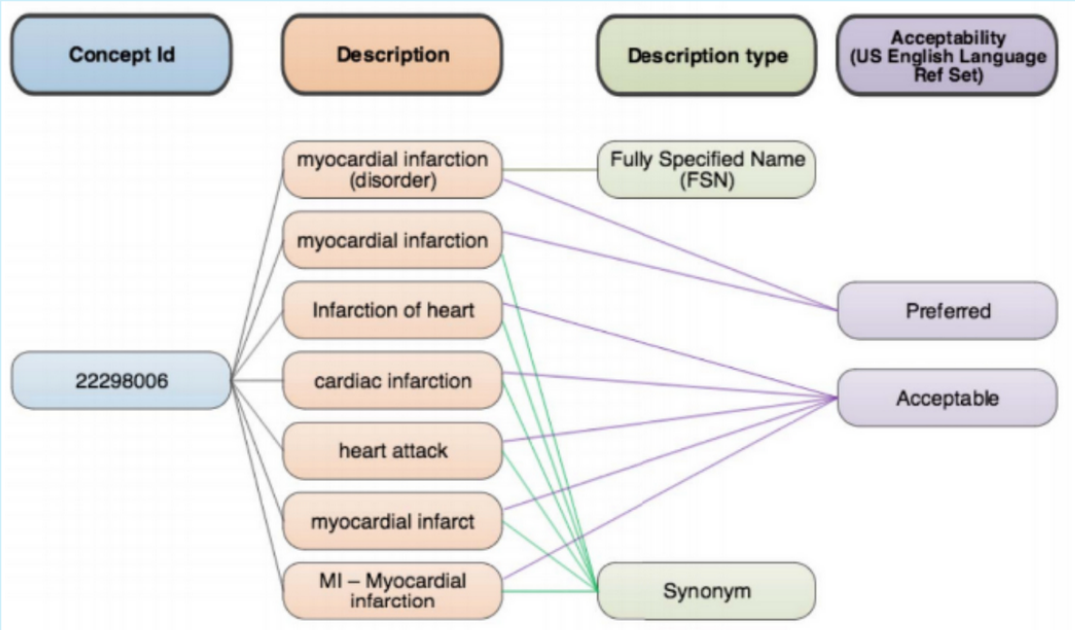

Fig. S1. Example of a SNOMED Clinical Terms ID (SCTID). The SCTID 22298006 has as Fully Specified Name (FSN) "myocardial infarction (disorder)", but it also known under different synonyms all pointing to the same concept. This procedure allowed us to identify which diagnoses point to the same medical concept. Figure reproduced from https://www.snomed.org/snomed-ct/five-step-briefing.

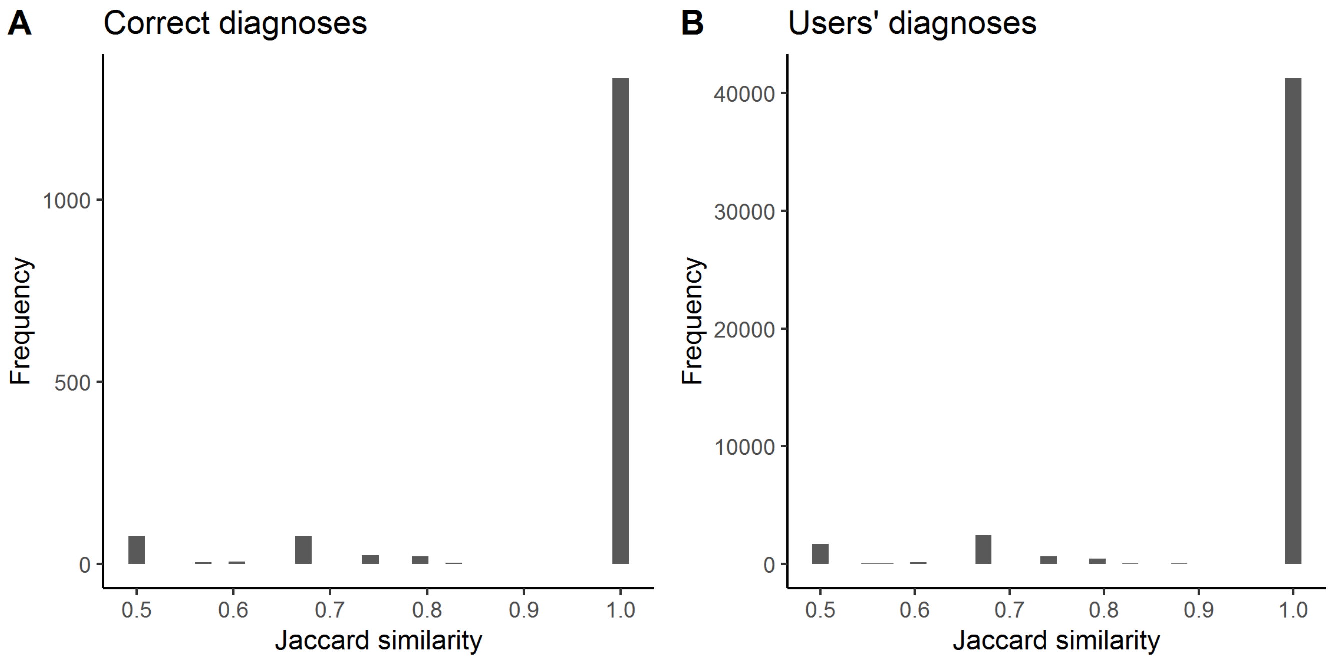

Fig. S2. The frequency distribution of the maximum Jaccard similarity values between the SNOMED CT terms and the (A) correct diagnoses and (B) users' diagnoses. For each (correct and user's) diagnosis, only the maximum Jaccard similarity value is shown. Correct diagnoses could be matched to a SCTID with a Jaccard similarity of 1 in 1,333 out of 1,572 cases. Users' diagnoses could be matched with a Jaccard similarity of 1 in 41,242 out of 47,772 cases. Only Jaccard similarity values of 0.5 and higher are shown—excluding 3 correct diagnoses and 854 users' diagnoses with a Jaccard similarity value below 0.5.

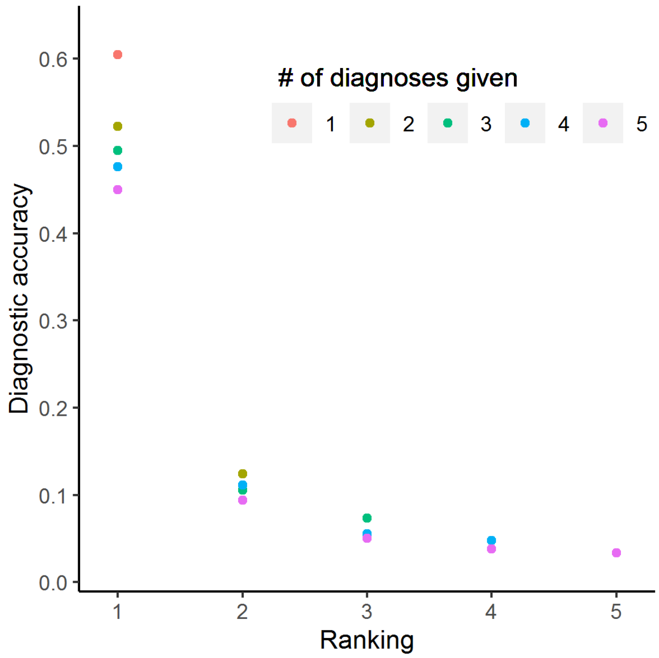

Fig. S3. Diagnostic accuracy of individual diagnoses as a function of the number of reported diagnoses by a user, and the ranking provided by the user. The likelihood that a given diagnosis was correct was highest when a user only gave one diagnosis (and by extension that diagnoses was ranked first). Users providing more than one diagnosis also had a relatively high likelihood that their first-ranked diagnosis was correct, but this likelihood quickly dropped for lower-ranked diagnoses.

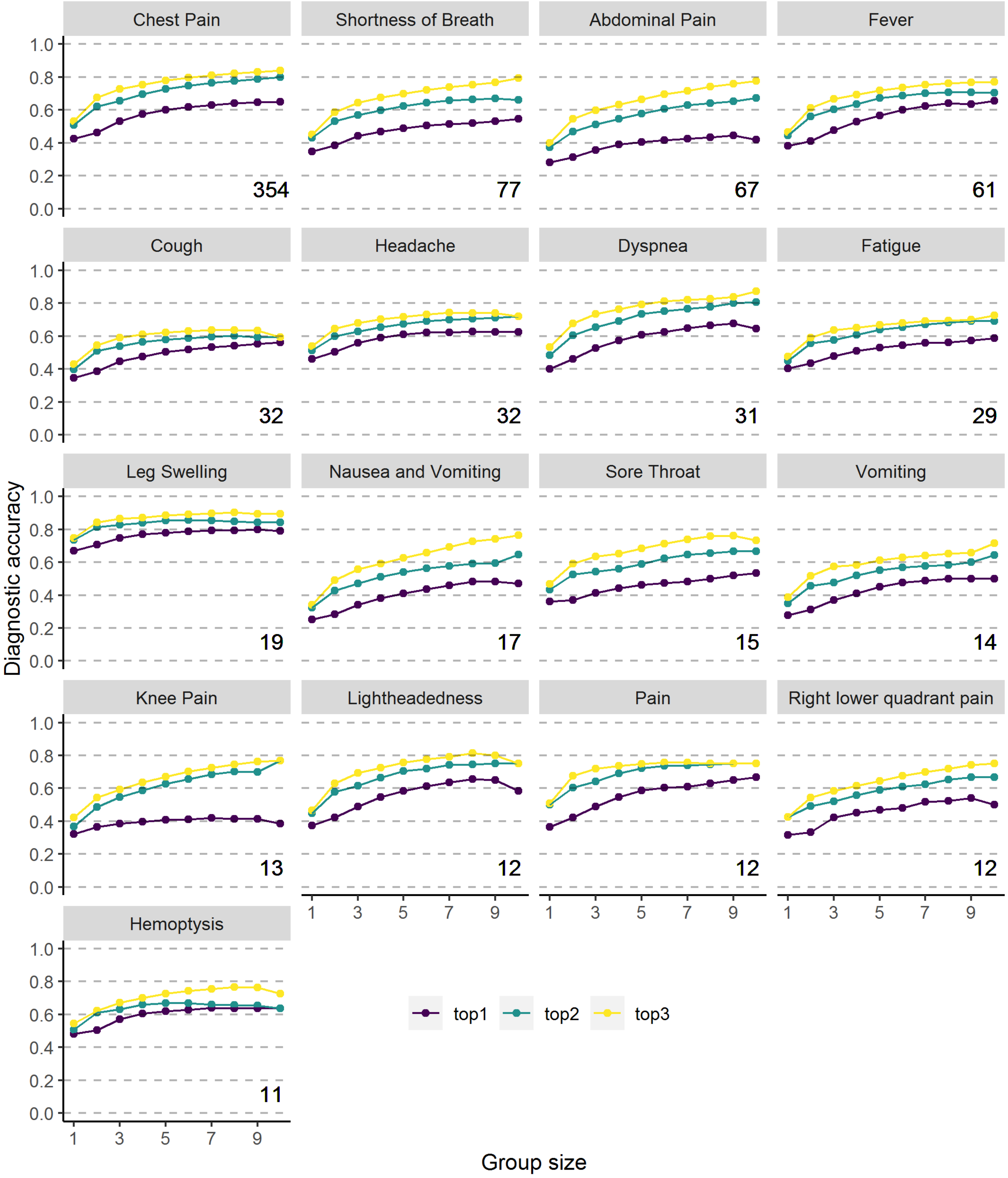

Fig. S4. Diagnostic accuracy of the aggregation procedure for different group sizes for different chief complaint of cases using the $1/r$ rule. Across chief complaints, increasing the number of group members increases the likelihood that the correct diagnosis is present in the top 1, top 2 or top 3 of the collective diagnosis. Numbers in the bottom right indicate the number of cases within that specialty. Medical specialties are ordered from highest to lowest number of cases. Only chief complaints with more than 10 cases are shown.

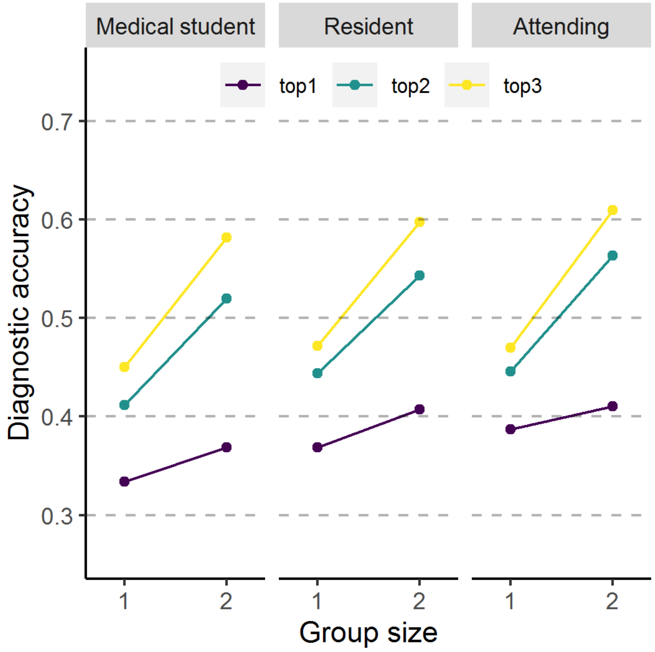

Fig. S5. Diagnostic accuracy of the aggregation procedure for small group sizes for different tenure levels of users only including cases that were rated by at least two medical students, residents and attending physicians (n = 450 cases). Across different tenure levels, increasing the number of group members increases the likelihood that the correct diagnosis is present in the top 1, top 2 or top 3 of the collective diagnosis.

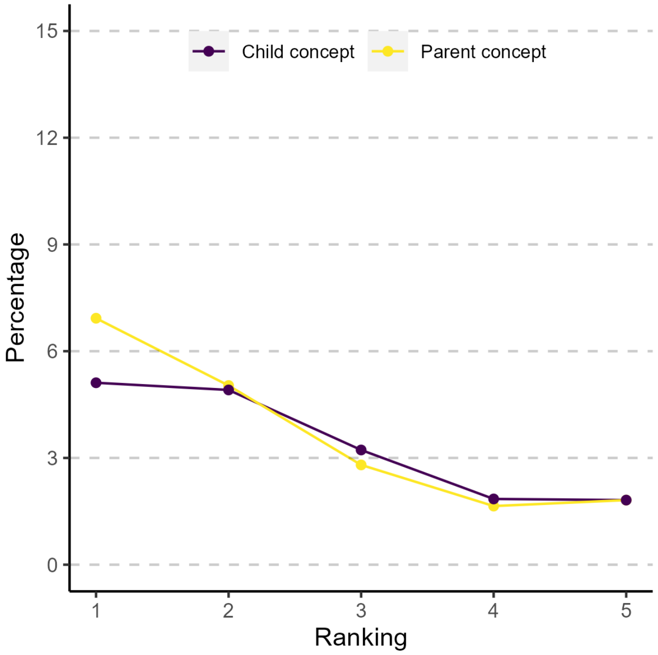

Fig. S6. The likelihood of a diagnosis being either a child or parent concept of the correct diagnosis as a function of the ranking provided by single users.

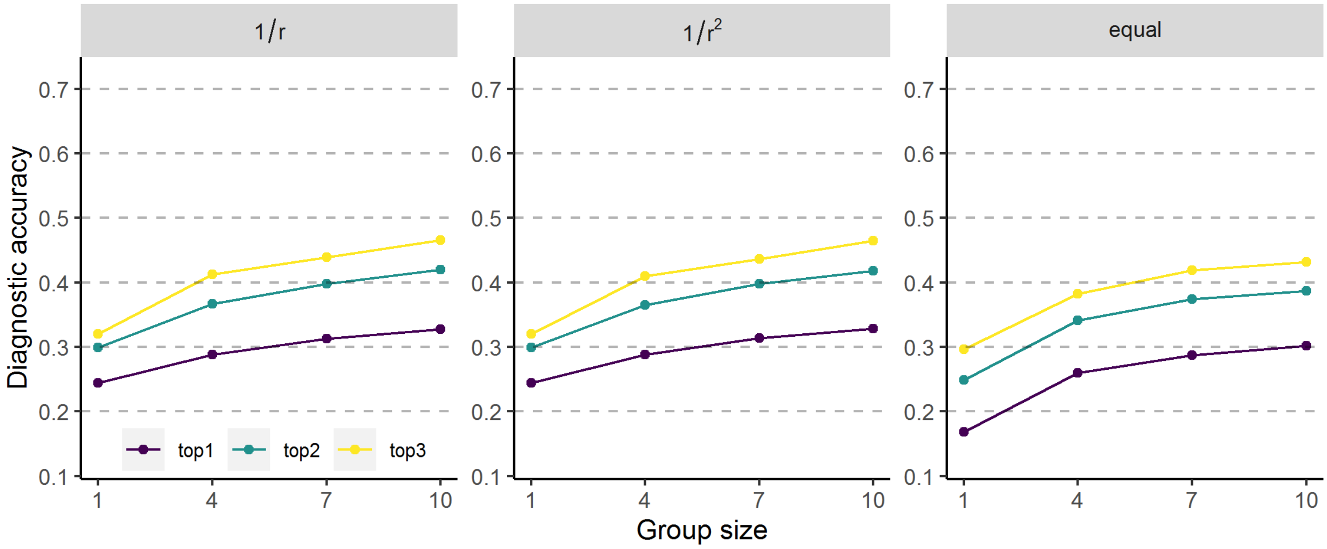

Fig. S7. Diagnostic accuracy of the aggregation procedure when using a Jaccard similarity of 0.6 as threshold. The individual and collective performance substantially dropped when using this threshold.

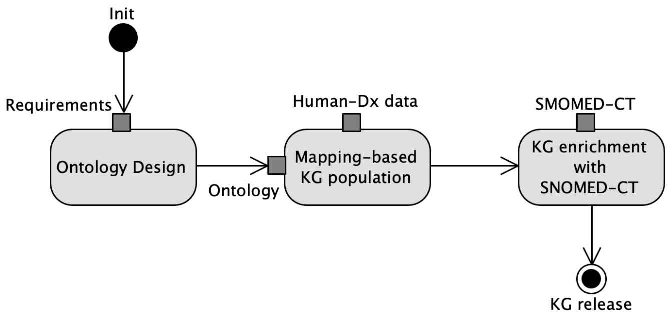

Fig. S8. Methodology adopted for knowledge graph (KG) construction. Activities are depicted as light-gray boxes, while the black circle and encircled black circle represent the initial and final nodes, respectively. The arrows among activities represent the direction of the workflow execution. The dark-gray boxes pinned on the activities identifies the objects required by the activities as input.

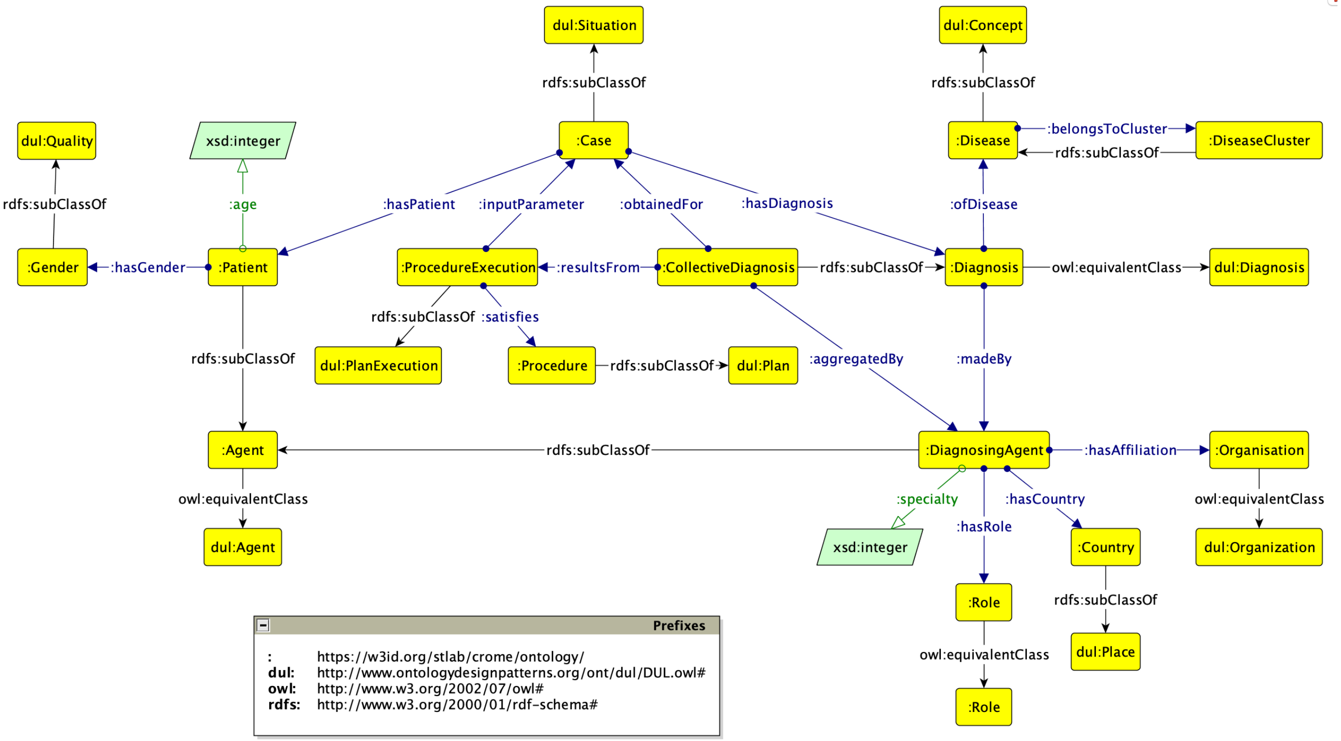

Fig. S9. Ontology. The ontology is depicted by using the Graphical Framework for OWL Ontologies (Graffoo) notation. In this notation the classes are represented as yellow rectangles, the datatypes as green parallelograms, the object properties as blue directed arrows, and the predicates as black directed arrows. The namespace adopted for defining ontology entities (i.e. classes and properties) is `https://w3id.org/stlab/crome/ontology/`, which is associated with the prefix `:` in the diagram.

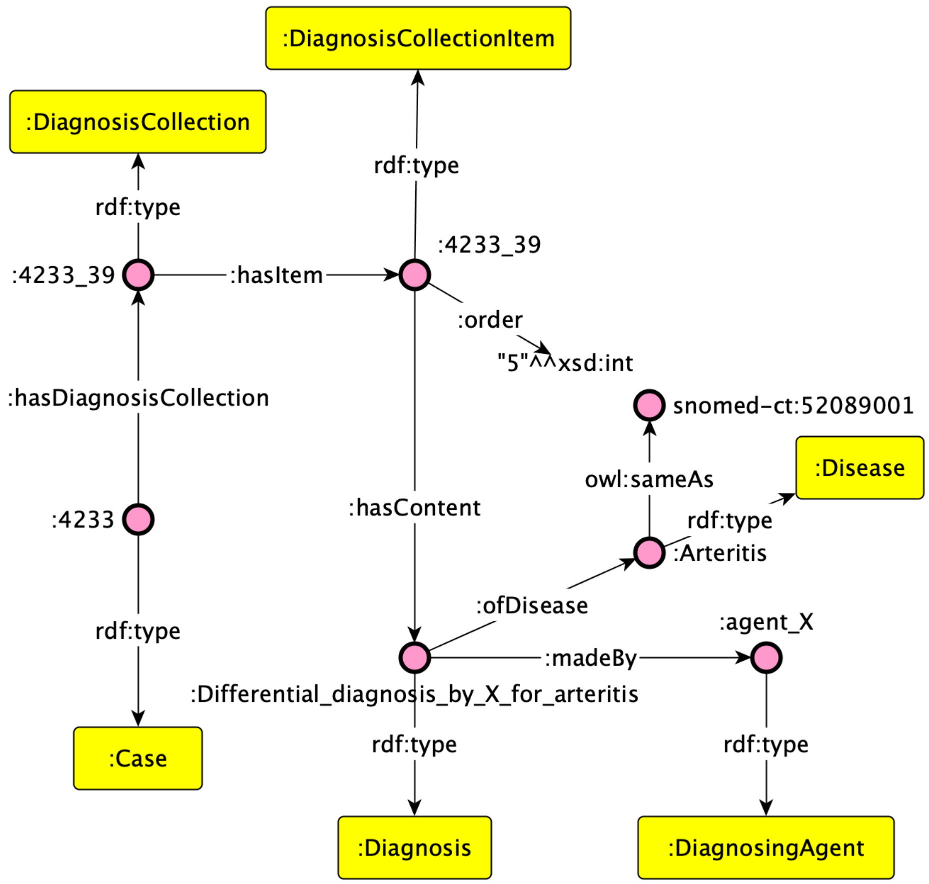

Fig. S10. Sample of the resulting KG. The sample KG is depicted by using the Graphical Framework for OWL Ontologies (Graffoo) notation. In this diagram instances are depicted as pink circles.

References

1. KA Spackman, KE Campbell, RA Côté, Snomed rt: a reference terminology for health care. in *Proceedings of the AMIA annual fall symposium.* (American Medical Informatics Association), p. 640 (1997).
2. K Donnelly, , et al., Snomed-ct: The advanced terminology and coding system for ehealth. *Stud. health technology informatics* **121**, 279 (2006).
3. PA Bonatti, S Decker, A Polleres, V Presutti, Knowledge graphs: New directions for knowledge representation on the semantic web (dagstuhl seminar 18371) in *Dagstuhl reports.* (Schloss Dagstuhl-Leibniz-Zentrum fuer Informatik), Vol. 8, (2019).
4. A Hogan, et al., Knowledge graphs. *Synth. Lect. on Data, Semantics, Knowl.* **12**, 1–257 (2021).
5. VA Carriero, et al., Pattern-based design applied to cultural heritage knowledge graphs. *Semantic Web* **12**, 313–357 (2021) DOI: 10.3233/SW-200422.
6. AG Nuzzolese, AL Gentile, V Presutti, A Gangemi, Conference linked data: The scholarlydata project. in *International Semantic Web Conference (2)*, Lecture Notes in Computer Science, eds. P Groth, et al. Vol. 9982, pp. 150–158 (2016).
7. A Gangemi, N Guarino, C Masolo, A Oltramari, L Schneider, Sweetening ontologies with dolce. in *EKAW*, Lecture Notes in Computer Science, eds. A Gómez-Pérez, VR Benjamins. (Springer), Vol. 2473, pp. 166–181 (2002).
8. A Dimou, et al., Rml: A generic language for integrated rdf mappings of heterogeneous data in *LDOW*, CEUR Workshop Proceedings, eds. C Bizer, T Heath, S Auer, T Berners-Lee. (CEUR-WS.org), Vol. 1184, (2014).
9. ACN Ngomo, S Auer, Limes - a time-efficient approach for large-scale link discovery on the web of data. in *IJCAI*, ed. T Walsh. (IJCAI/AAAI), pp. 2312–2317 (2011).
